# Comparison of conventional Papanicolaou cytology samples with liquid-based cervical cytology samples from women in Pernambuco, Brazil

**DOI:** 10.1590/1414-431X20154252

**Published:** 2015-07-31

**Authors:** M.O.L.P. Costa, S.A. Heráclio, A.V.C. Coelho, V.L. Acioly, P.R.E. Souza, M.T.S. Correia

**Affiliations:** 1 Laboratório Central de Saúde Pública do Estado de Pernambuco, Recife, PE, Brasil; 2 Departamento de Bioquímica, Universidade Federal de Pernambuco, Recife, PE, Brasil; 3 Departamento de Biologia, Universidade Federal Rural de Pernambuco, Recife, PE, Brasil; 4 Departamento de Genética, Universidade Federal de Pernambuco, Recife, PE, Brasil

**Keywords:** Papanicolaou cytology, Liquid-based cytology, ThinPrep

## Abstract

In the present study, we compared the performance of a ThinPrep cytological method with the conventional Papanicolaou test for diagnosis of cytopathological changes, with regard to unsatisfactory results achieved at the Central Public Health Laboratory of the State of Pernambuco. A population-based, cross-sectional study was performed with women aged 18 to 65 years, who spontaneously sought gynecological services in Public Health Units in the State of Pernambuco, Northeast Brazil, between April and November 2011. All patients in the study were given a standardized questionnaire on sociodemographics, sexual characteristics, reproductive practices, and habits. A total of 525 patients were assessed by the two methods (11.05% were under the age of 25 years, 30.86% were single, 4.4% had had more than 5 sexual partners, 44% were not using contraception, 38.85% were users of alcohol, 24.38% were smokers, 3.24% had consumed drugs previously, 42.01% had gynecological complaints, and 12.19% had an early history of sexually transmitted diseases). The two methods showed poor correlation (*k*=0.19; 95%CI=0.11–0.26; P<0.001). The ThinPrep method reduced the rate of unsatisfactory results from 4.38% to 1.71% (χ^2^=5.28; P=0.02), and the number of cytopathological changes diagnosed increased from 2.47% to 3.04%. This study confirmed that adopting the ThinPrep method for diagnosis of cervical cytological samples was an improvement over the conventional method. Furthermore, this method may reduce possible losses from cytological resampling and reduce obstacles to patient follow-up, improving the quality of the public health system in the State of Pernambuco, Northeast Brazil.

## Introduction

Cervical carcinoma is the second most frequent cancer type and the third leading cause of death by cancer in women worldwide (1). Thus, it becomes an important public health problem. According to the latest global estimates, there were 527,000 new cases and 265,000 cervical cancer related deaths in 2012, with 85% of total cases located in developing countries. The absence or low effectiveness of prevention programs (cytological screening) is singled out as the likely cause of the high incidence of cervical cancer in these countries (1).

As is widely known, human papilloma virus (HPV) infection is a prerequisite necessary but not sufficient cause of cervical lesions, implying that the combination of host, bacterial, environmental and genetic factors, along with persistent infection with high-risk HPV strains, may be considered as cofactors rather than independent factors. The main prevention tool against cervical cancer is cytological screening, and vaccination against HPV, which has high efficacy for prevention of HPV infection and its associated lesions; but barriers to vaccination include costs, limited vaccine availability, and lack of vaccine awareness (2).

In Brazil, 15,590 new cases of cervical cancer were expected in 2014, with an estimated risk of 15.33 cases for every 100,000 women. Unlike other types of human cancers, cervical cancer is a preventable disease, due to its slow progression, with a long period from the development of precursor lesions to the emergence of neoplasia (3).

The Papanicolaou cytological examination (conventional Pap test, CP), developed by the Greek doctor Geórgios Papanicolaou in 1941 as a tool for early detection of cervical cancer, is the main strategy used in control programs of cervical cancer. In Brazil, the Ministry of Health has determined that the CP should be performed primarily in women aged 25 to 64 years (2).

The CP is considered an efficient and easy-to-apply methodology, as it has the ability to identify precursor lesions of cervical cancer while they are still treatable (2). However, despite its well-known methodology, the CP has high rates of false-negatives due to its oscillation in sensitivity (4), and those rates can vary from 2% to 50% (2,5,6). In a meta-analysis study conducted by Fahey et al. (7), sensitivity of the CP was found to be 58% (ranging from 11% to 99%), with a specificity of 68% (ranging from 14% to 97%).

In the 1990s, a new methodology was developed for the collection and preparation of cervical cytological samples for screening, a liquid-based cytology called the ThinPrep¯ Pap test (Cytyc Corporation, USA). Approved in 1996 by the United States Food and Drug Administration, ThinPrep was introduced as an alternative to using the conventional method, with the purpose of improving the screening of atypical cells, cervical cancer, or its precursor lesions [low-grade squamous intraepithelial lesions (LSIL) and high-grade squamous intraepithelial lesions (HSIL)]. The aim was to improve sensitivity, because it permits the use of a monolayer of cells to facilitate the diagnosis by the cytopathologist, with better cellular preservation and the possibility to carry out molecular biology testing, making possible, for example, HPV and *Chlamydia trachomatis* DNA detection (4,8-12). This technique has been widely adopted and is gradually replacing the CP in control programs of cervical cancer in some countries (13,14).

There is sufficient evidence that the ThinPrep method reduces the proportion of unsatisfactory samples. However, there are still doubts about its effectiveness, as is also true for the conventional method, in the early detection of cervical cancer (15-17).

The State of Pernambuco (Northeast Brazil) stands out because of its high rates of unsatisfactory tests: of 185 municipalities, 77 (41.62%) had rates of unsatisfactory results above 5% during the year 2013 (18). The Central Public Health Laboratory of the State of Pernambuco (LACEN-PE) is the laboratory where a great number of statewide samples are processed and examined. Thus, the present study was conducted to compare the rates of cytopathological changes and unsatisfactory results of the two methodologies (CP cytology and liquid-based ThinPrep cytology) in cervical samples of women served by the Public Health Units of the State of Pernambuco and analyzed by LACEN-PE, and we also evaluated clinical, biological, and sociodemographic characteristics of these patients.

## Material and Methods

A population-based cross-sectional study was conducted with women aged between 18 and 64 years, attended by spontaneous demand at Public Health Units of the State of Pernambuco, Brazil, during the period between April and November 2011. These units make up 63.24% of the public health care system in the State of Pernambuco, which is divided into 185 municipalities with basic health units in five regions as follows: Recife (19 units), Limoeiro (31 units), Palmares (22 units), Caruaru (32 units), and Arcoverde (13 units). On average these units serve 60 patients each week, and more than 70% of them are of low socioeconomic status.

Cervical smear samples were collected by various professionals (nurses, cytotechnologists, and cytopathologists) at different basic health centers. All cytological slides were then referred to LACEN-PE, a public reference center for female genital diseases in Pernambuco, Brazil, where the study was carried out. The slides were screened by local cytotechnologists who stained them for the Papanicolaou technique, and were classified by one of the 16 local cytopathologists according to international norms of standardization (Bethesda System 2001/adapted by the Brazilian Society of Cytopathology). Patients who had undergone radiation treatment or chemotherapy for invasive cervical neoplasia and/or who had been subjected to oncotic cytology collection within the last 3 months before recruitment were excluded from the study.

A double-blind study was designed as follows: paired specimens were subjected to both CP and ThinPrep, permitting the evaluation of the two methodologies. The collection of cervical-vaginal material for the method of liquid-based preparation was similar to the collection of material for the conventional method, changing only the instrument used and the number of turns made for obtaining the sample.

In one-half of the samples, the material was initially transferred to the conventional frosted slide with an endocervical brush and Ayres spatula and fixed with polyethylene glycol spray, and then new samples were collected and agitated in the supernatant of the liquid medium for the ThinPrep Pap test, according to the manufacturer's instructions (15). In the other half, the endocervical brush was first agitated in the supernatant of the liquid medium, and then immediately a new brush and spatula were used for conventional cytology. Samples were transported at the end of the day to the LACEN-PE, where they were kept under refrigeration (between 5 and 8°C) awaiting analysis for a maximum of 1 month.

Information on sociodemographics, sexual characteristics, reproductive practices, and habits of patients (such as smoking, alcohol consumption, and drug use) was obtained from all patients on a standardized questionnaire.

The study was approved in advance by the Ethics Committee for Research of the Universidade Federal de Pernambuco (#105/09-CCS), and all patients signed an informed consent form and filled out the questionnaires.

The sample size was calculated using the STATCALC program of Epi-Info 3.5 for Windows (USA), based on prevalence data from the literature. For statistics, the chi-square test of association (Pearson) was used at a significance level of 5%, and the kappa and McNemar tests were also used to better evaluate discrepancies using the statistical software (Epi-Info version 5.0 or higher) with double-entry.

Cohen's kappa test for agreement between paired data measurements was performed to assess the level of agreement between the results of the two methodologies applied to the same samples (paired data). If a level of low agreement was observed, then a McNemar test was applied to verify which results were responsible for the low agreement. Therefore, we compared the sensitivity of the ThinPrep method taking conventional cytology results in the same individual as a reference.

## Results

In this study, cervical samples from 525 women were included and analyzed at the LACEN-PE. The distribution of sociodemographics, sexual characteristics, reproductive practices, and habits of patients is shown in Table 1. This sample consisted of a majority of women older than 25 years (86.7%), with elementary-level schooling (52.2%; 8 years or less of study), predominantly from urban areas (71.1%). Regarding ethnicity, 47.6% self-reported being mulatto. In addition, the majority of the women reported being married or otherwise in a stable relationship (69.1%). The majority of women reported having five or less total number of sexual partners (93.5%). Although previous sexually transmitted infections (STI) episodes were not reported for the women before being recruited for the present study (87.8%), some had previously complained about genital discharge, bleeding, or rashes (42.1%). Regarding contraceptive methods, some women reported not using any method whatsoever (31.8%). Other women reported surgical sterilization (tubal ligation: 31.8%, hysterectomy: 6.3%). Only 5.3% of the women reported the frequent use of condoms, and 8.6% used a combination of oral contraceptive pills. Only 0.8% of the women reported being in menopause.

**Table:**
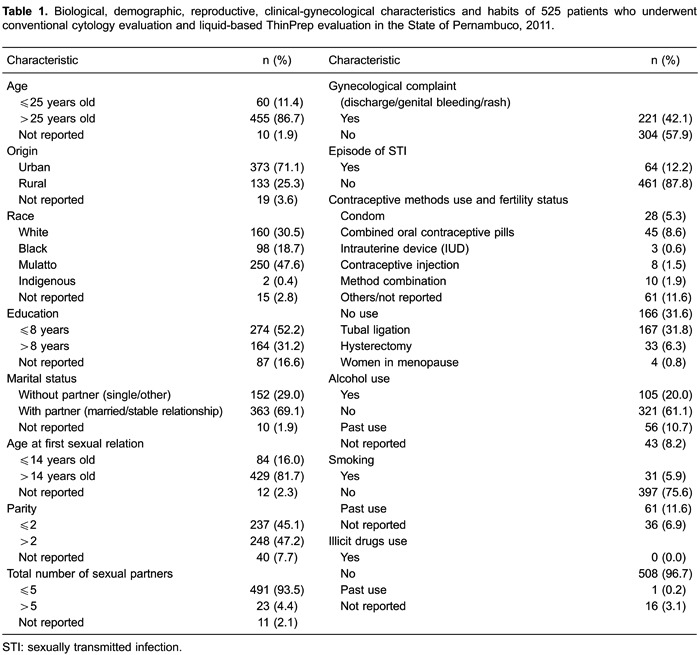

It was observed that 5.9% were smokers (11.6% reported past use), 20.0% reported frequent alcohol use, and only one woman (0.2%) reported past use of illicit drugs (marijuana).

The results of the diagnostic interpretations between the two methodologies, ThinPrep and CP, are shown in Table 2. Evaluation of agreement between methodologies through Cohen's kappa test resulted in a measure of weak agreement between the two methods, kappa=19%. The low level of agreement was due to an increase in the percentage of normal diagnoses and altered diagnoses using the ThinPrep methodology (from 34.7% to 48.8% and from 2.4% to 3.0%, respectively) and a reduction in the number of inflammatory and unsatisfactory diagnoses (from 58.5% to 46.5% and from 4.4% to 1.70%, respectively; P<0.01 for both analyses; Table 2).

**Table:**
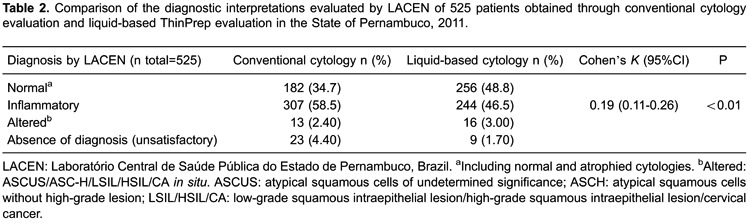

Under a dichotomous classification (satisfactory or unsatisfactory), we observed that the ThinPrep method was better than conventional cytology for diagnostic definition, because all 23 samples with unsatisfactory results by CP had defined diagnostics using the ThinPrep protocol. In other words, the conventional test had 4.4% (23/525) unsatisfactory results, *vs* only 1.7% (9/525) unsatisfactory results from the ThinPrep test (McNemar χ^2^=5.28, P=0.02 for both analyses; Table 3). Finally, no significant differences were observed in the detection of altered cytology between the tests carried out. A total of 13 samples were detected by CP (2.47%) and 16 by ThinPrep (3.04%, χ^2^=0.24; P=0.63; Table 4).

**Table:**
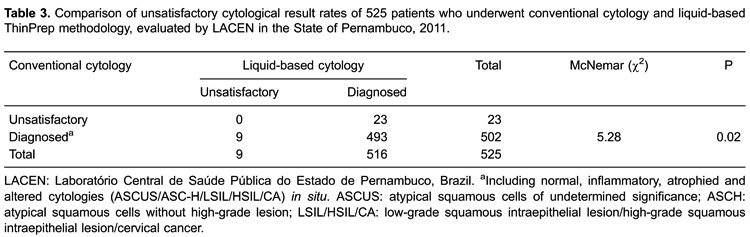

**Table:**
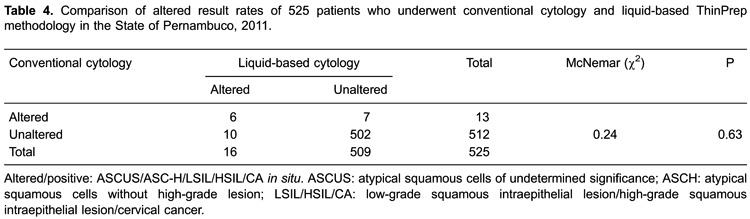

## Discussion

Bezerra et al. (19) and Amaral et al. (20) relate early onset of sexual activity, multiple sexual partners, use of oral contraceptives, smoking, nutritional deficiency, and immunological state as important risk factors for the development of neoplastic lesions. Analyzing the sociodemographics, sexual characteristics, reproductive practices, and habits of patients (such as smoking, alcohol consumption, and drug use), it was observed that users of the Public Health System in the State of Pernambuco (SUS-PE) are young women, of a low educational level, who use few methods of protection, both from the reproductive point of view and for prevention of STIs. Dunne et al. (21) found a higher prevalence of HPV infection among women who have lower education, are unmarried, belong to certain racial or ethnic groups, and have lower socioeconomic status. We speculate that the Public Health Service outreach for cervical cancer screening is greater among women over 25 years of age and among women living in a geographic area with greater access to public services that promote prevention and recovery of health.

Several authors have demonstrated and confirmed the advantages of ThinPrep in relation to CP for cervical exams (8,22-25). ThinPrep has been referred to as the method of superior performance because it provides better cellular representation, with increased sensitivity for the detection of lesions, compared with conventional methodology (5,8,14,24-28). Several authors have shown that this greater diagnostic sensitivity applies to high-grade and glandular lesions (26-28).

Other authors note that ThinPrep allows better preservation and cellular disposition, allowing for better diagnostic interpretation, due to reduction of the presence of mucus, inflammatory exudates, and erythrocytes; reduction in reading time, in addition to enabling the processing of additional samples without the need to call in the patient for new material collection; and allowing the use of residual samples for molecular biology testing of viruses such as HPV (4,26) and of various other associated pathogenic organisms (e.g., *C. trachomatis*, *Neisseria gonorrhoeae*, etc.). However, the disadvantages identified are related to the higher cost and the need to train professionals in the new technique.

In most studies comparing the two methods, ThinPrep increases the quality of the results by reducing the number of cases classified as unsatisfactory (27-32). Cheung et al. (31) found a reduction in the rate of unsatisfactory results with the ThinPrep method, from 0.48% to 0.32%. Similarly, in the current study, there was a reduction in the rate of unsatisfactory results from 4.4% to 1.70% (P<0.01; Table 2).

In the present study, the percentage of inflammatory results with the CP method was 58.47% *vs* 46.47% with ThinPrep. However, there was an increase in the detection of altered cells by ThinPrep (3.04% *vs* 2.47%). Khalbuss et al. (33) detected a greater number of blood cells and inflammatory cells using CP. These data can be explained by considering that, for liquid-based ThinPrep, because the reading area is reduced by up to 81% and interference that normally obscures the samples is eliminated, there is an increase of about 50% in reading time and improvements of up to 73% in lab productivity (8,23).

Data from the literature were controversial when reporting satisfactory diagnosis rate comparisons between CP and ThinPrep, although some studies did not show statistically significant differences (13,31-33). Khalbuss et al. (33) and Cheung et al. (31) did not detect any significant difference between the two methods. In contrast, Coste et al. (34) detected an increase in satisfactory results using CP (91%) compared with ThinPrep (87%). The same was observed by Stabile et al. (8), where quality was measured according to the presence of elements of the squamocolumnar junction. These researchers detected an increase in the number of diagnoses with CP compared with liquid cytology (93% *vs* 84%, respectively). Corroborating with these last two studies, our results showed an increase in the percentage of satisfactory results using the ThinPrep method (98.3% *vs* 95.6%; P=0.02).

Agreement between the ThinPrep and CP methods, in a meta-analysis conducted by Abulafia et al. (10) using 17 articles selected from the literature in the period between 1990 and 2002, found agreement among 89% of the cases based on a classification with five levels of diagnosis (negative, atypical, LSIL, HSIL, and carcinoma).

In the present analysis, despite the increase in the number of cytopathological changes detected by ThinPrep (3.04% *vs* 2.47%), this difference was not significant (P=0.63; Table 4). Similarly, Davey et al. (35) conducted a meta-analysis study of 56 studies and did not detect any difference between the performances of the two techniques. Jesdapatarakul et al. (36) also did not observe any significant improvement in the diagnosis when comparing both methods. In contrast, Stabile et al. (8) detected 3% atypical diagnoses with CP *vs* 10% with liquid-based cytology. Cheung et al. (31) detected an increase in the number of diagnoses by ThinPrep compared with CP (3.74% *vs* 3.19% for atypical squamous cells of undetermined significance (ASCUS) and 1.67% *vs* 1.01% for LSIL).

A limiting factor of this study is that it was not possible to assess the specificity and sensitivity of the techniques analyzed, because not all the patients were evaluated by histopathology, which is used as the gold standard in cytology. However, results of some previous studies regarding the sensitivity and specificity of the conventional and liquid-medium methods were summarized in a meta-analysis conducted by Abulafia et al. (10). In that study, they found 68% general sensitivity for the CP and 76% for ThinPrep. However, the difference was statistically significant in only two studies. The same was observed in relation to specificity, which was 79% for CP and 86% for ThinPrep, a difference that was not significant in most cases. In another study, conducted by Coste et al. (34), the sensitivity of conventional cytology ranged from 57% to 74%, while liquid-based cytology ranged from 61% to 73%. The specificity ranged from 91% to 96% for CP and 90% to 95% for ThinPrep. However, diagnoses using liquid-based cytology reported more ASCUS-type abnormalities. Similarly, Arbyn et al. (37), following a systematic review and meta-analysis of 109 studies of various designs, noted that liquid-based cytology did not provide significant differences in sensitivity and specificity.

It is important to stress that the collection of material was performed by various professionals in different health centers, which may be related to false-negative results. Even though guidelines for such procedures were applied to the laboratories, there was no way to ensure effective standardization of the quality of the process when executed manually. According to recommendations in the Executive Summary of the National Program for Control of Cervical Cancer in 2010 (38), published by the Pan-American Health Organization, in order to maintain quality control standards, a laboratory must have a minimum production of 15,000 exams/year. In Brazil, among the laboratories that provided services for Public Health Units in 2008, only 15% of a total of 1116 laboratories presented production above this threshold.

Given the results obtained from the population of this study, it is possible to conclude that liquid-based cytology offered an improvement in cytological diagnosis and contributed to a decrease in the number of unsatisfactory results in the reports from the Public Health network in the State of Pernambuco, Brazil. Our results show that around 5% of the women would not have a correct diagnosis through CP. In principle, 5% seems low, but considering that Pernambuco has a high prevalence of HPV infection, it is possible that a majority of this 5% of women may already have lesions or even altered cytology, but their clinicians would not know because of unsatisfactory methodologies.

Thus, although liquid-based cytology is more expensive, its widespread introduction to the routine of the Pernambuco public health network will allow the establishment of standards in collection, preparation, and staining of samples that will guarantee an improvement in the quality of testing and diagnostics, as well as reduce possible losses from cytological repetition, and support additional investigation of STIs in the population of this area, in the State of Pernambuco, Brazil.

## References

[1] WHO (World Health Organization) (2014). Human papillomavirus (HPV) and cervical cancer.

[2] Tavares SBN, Amaral RG, Manrique EJC, Sousa NLA, Albuquerque ZBP, Zeferino LC (2007). Controle de qualidade em citopatologia cervical: Revisão
de literatura. Rev Bras Cancer.

[3] Ministério da Saúde (2015). Estimativa 2014 - Incidência de Câncer no Brasil..

[4] Bernstein SJ, Sanchez-Ramos L, Ndubisi B (2001). Liquid-based cervical cytologic smear study and
conventional Papanicolaou smears: a metaanalysis of prospective studies comparing
cytologic diagnosis and sample adequacy. Am J Obstet Gynecol.

[5] Bergeron C, Masseroli M, Ghezi A, Lemarie A, Mango L, Koss LG (2000). Quality control of cervical cytology in high-risk women.
PAPNET system compared with manual rescreening. Acta Cytol.

[6] Mattosinho de Castro Ferraz Mda G, Dall’Agnol M, di Loreto C, Pirani WM, Utagawa ML, Pereira SMM (2005). 100% rapid rescreening for quality assurance in a
quality control program in a public health cytologic laboratory. Acta Cytol.

[7] Fahey MT, Irwig L, Macaskill P (1995). Meta-analysis of Pap test accuracy. Am J Epidemiol.

[8] Stabile SA, Evangelista DH, Talamonte VH, Lippi UG, Lopes RG (2012). Comparative study of the results from conventional
cervico-vaginal oncotic cytology and liquid-based cytology. Einstein.

[9] Takei H, Ruiz B, Hicks J (2006). Cervicovaginal flora. Comparison of conventional pap
smears and a liquid-based thin-layer preparation. Am J Clin Pathol.

[10] Abulafia O, Pezzullo JC, Sherer DM (2003). Performance of ThinPrep liquid-based cervical cytology
in comparison with conventionally prepared Papanicolaou smears: a quantitative
survey. Gynecol Oncol.

[11] Bidus MA, Maxwell GL, Kulasingam S, Rose GS, Elkas JC, Chernofsky M (2006). Cost-effectiveness analysis of liquid-based cytology and
human papillomavirus testing in cervical cancer screening. Obstet Gynecol.

[12] Ronco G, Segnan N, Giorgi-Rossi P, Zappa M, Casadei GP, Carozzi F (2006). Human papillomavirus testing and liquid-based cytology:
results at recruitment from the new technologies for cervical cancer randomized
controlled trial. J Natl Cancer Inst.

[13] Hoelund B (2003). Implementation of liquid-based cytology in the screening
programme against cervical cancer in the County of Funen, Denmark, and status for
the first year. Cytopathology.

[14] Payne N, Chilcott J, McGoogan E (2000). Liquid-based cytology for cervical
screening. Cytopathology.

[15] USA Public Health Service (2014). FDA DoHaHS. Center for Devices and Radiological Health (CDRH). Approval
letter for the ThinPrep¯2000 System. Approval Application No. P950039 - FDA.
Rockville, MD, 1996.

[16] Girianelli VR, Santos Thuler LC (2007). Evaluation of agreement between conventional and
liquid-based cytology in cervical cancer early detection based on analysis of
2,091 smears: experience at the Brazilian National Cancer
Institute. Diagn Cytopathol.

[17] Campagnoli EB, Sandrin R, Braosi AP, Lima AA, França BH, Machado MA (2005). Citologia em base líquida - uma nova opção para o
diagnóstico de lesões bucais. Rev Bras Patol Oral.

[18] Ministério da Saúde, DATASUS (2014). Informações de Saúde. Epidemiológicas e morbidades. Câncer de
colo de útero e mama.

[19] Bezerra SJS, Gonçalves PC, Franco ES, Pinheiro AKB (2005). Perfil de mulheres portadoras de lesões cervicais por
HPV quanto aos fatores de risco para câncer de colo uterino. DST - J Bras Doenças Sex Transm.

[20] Amaral RG, Manrique EJ, Guimaraes JV, Sousa PJ, Mignoli JR, Xavier AF (2008). [Influence of adequacy of the sample on detection of the
precursor lesions of the cervical cancer]. Rev Bras Ginecol Obstet.

[21] Dunne EF, Unger ER, Sternberg M, McQuillan G, Swan DC, Patel SS (2007). Prevalence of HPV infection among females in the United
States. JAMA.

[22] Pereira SMM, Utagawa ML, Pittoli JE, Aguiar LS, Maeda MYS, Longatto A (2003). Avaliação da celularidade citológica em preparados de
base líquida. Ver Inst Adolfo Lutz.

[23] Dias EP, Milagres A, Santos JB, Valladares CP, Souza ACB, Pinheiro RS (2008). Estudo comparativo de raspados orais submetidos è
técnica de citologia em meio líquido e citopatologia convencional. J Bras Patol Med Lab.

[24] Alves AV, Bibbo M, Schmitt FC, Milanezi F, Longatto A (2003). Comparison of manual and automated methods of
liquid-based cytologya: a morphologic study. Acta Cytol.

[25] Schledermann D, Ejersbo D, Hoelund B (2004). Significance of atypia in conventional Papanicolaou
smears and liquid-based cytology: a follow-up study. Cytopathology.

[26] Baker JJ (2002). Conventional and liquid-based cervicovaginal cytology: a
comparison study with clinical and histologic follow-up. Diagn Cytopathol.

[27] Grace A, McBrearty P, Troost S, Thornhill M, Kay E, Leader M (2002). Comparative study: conventional cervical and ThinPrep
Pap tests in a routine clinical setting. Cytopathology.

[28] Beerman H, van Dorst EB, Kuenen-Boumeester V, Hogendoorn PC (2009). Superior performance of liquid-based versus conventional
cytology in a population-based cervical cancer screening program. Gynecol Oncol.

[29] Weintraub J, Morabia A (2000). Efficacy of a liquid-based thin layer method for
cervical cancer screening in a population with a low incidence of cervical
cancer. Diagn Cytopathol.

[30] Bernstein SJ, Sanchez-Ramos L, Ndubisi B (2001). Liquid-based cervical cytologic smear study and
conventional Papanicolaou smears: a metaanalysis of prospective studies comparing
cytologic diagnosis and sample adequacy. Am J Obstet Gynecol.

[31] Cheung AN, Szeto EF, Leung BS, Khoo US, Ng AW (2003). Liquid-based cytology and conventional cervical smears:
a comparison study in an Asian screening population. Cancer.

[32] Anschau F, Gonçalves M (2006). Citologia cervical em meio líquido versus citologia
convencional. Femina.

[33] Khalbuss WE, Rudomina D, Kauff ND, Chuang L, Melamed MR (2000). SpinThin, a simple, inexpensive technique for
preparation of thin-layer cervical cytology from liquid-based specimens: data on
791 cases. Cancer.

[34] Coste J, Cochand-Priollet B, de Cremoux P, Le Gales C, Cartier I, Molinie V (2003). Cross sectional study of conventional cervical smear,
monolayer cytology, and human papillomavirus DNA testing for cervical cancer
screening. BMJ.

[35] Davey E, Barratt A, Irwig L, Chan SF, Macaskill P, Mannes P (2006). Effect of study design and quality on unsatisfactory
rates, cytology classifications, and accuracy in liquid-based versus conventional
cervical cytology: a systematic review. Lancet.

[36] Jesdapatarakul S, Tangjitgamol S, Nguansangiam S, Manusirivithaya S (2011). Liqui-Prep(R) versus conventional Papanicolaou smear to
detect cervical cells abnormality by split-sample technique: a randomized
double-blind controlled trial. Diagn Cytopathol.

[37] Arbyn M, Bergeron C, Klinkhamer P, Martin-Hirsch P, Siebers AG, Bulten J (2008). Liquid compared with conventional cervical cytology: a
systematic review and meta-analysis. Obstet Gynecol.

[38] Ministério da Saúde (2014). Plano de ação para redução da incidência e mortalidade por
câncer do colo do útero. Sumário executivo. Programa nacional de controle do
câncer do colo do útero..

